# Supplementary material for: P-TRAP: a Panicle Trait Phenotyping tool
Source: BMC Plant Biol. 2013 Aug 29;13:122. doi: 10.1186/1471-2229-13-122 (PMC3848748; doi:10.1186/1471-2229-13-122)
Supplement: Additional file 7 — P-TRAP source code. The Netbeans project that contains the source code of the application. [file 1471-2229-13-122-S7.zip › javadoc.zip!/javadoc/org-bioalg-graphprocessor/org/bioalg/graphprocessor/package-frame.html]

org.bioalg.graphprocessor (GraphProcessor)


# org.bioalg.graphprocessor

## Classes

- BranchModel
- Edge
- EdgeModel
- Graph
- GraphModel
- Path
- Vertex

## Enums

- Edge.EDGE\_TYPE
- GraphModel.GraphErrors
- Vertex.VERTEX\_TYPE
